# Acquisition time for functional near-infrared spectroscopy resting-state functional connectivity in assessing autism

**DOI:** 10.1117/1.NPh.9.4.045007

**Published:** 2022-11-30

**Authors:** Xiaoyin Wu, Fang Lin, Tingzhen Zhang, Huiwen Sun, Jun Li

**Affiliations:** a South China Normal University, South China Academy of Advanced Optoelectronics, Guangzhou, China; b South China Normal University, Key Lab for Behavioral Economic Science and Technology, Guangzhou, China

**Keywords:** resting-state functional connectivity, functional near-infrared spectroscopy, signal acquisition time, autism spectrum disorder, differentiation

## Abstract

**Significance:**

Resting state functional connectivity (RSFC) can be used to assess autism spectrum disorder (ASD). Measuring RSFC usually takes 5 to 10 min, during which children with ASD may have difficulty keeping their heads motionless. Therefore, a short acquisition time for RSFC would make clinical implementation more feasible.

**Aim:**

To find a suitable acquisition time necessary for measuring RSFC with functional near-infrared spectroscopy (fNIRS) for the differentiation between children with ASD and typically developing (TD) children.

**Approach:**

We used fNIRS to record the spontaneous hemodynamic fluctuations from the bilateral temporal lobes of 25 children with ASD and 22 TD children. The recorded signals were truncated into several segments with different time windows, and then the homotopic RSFC was computed for each of these segments and compared between the two groups.

**Results:**

We observed even in a very short time duration of 0.5 min, the RSFC had already existed a significant difference between the two groups, and 2.0 min might be the minimal time required for measuring RSFC for accurate differentiation between the two groups.

**Conclusions:**

The fNIRS-RSFC acquired even in a short time, e.g., 2.0 min, might be a reliable feature for the differentiation between children with ASD and TD children.

## 1 Introduction

Autism spectrum disorder (ASD) is a pervasive neurodevelopmental disorder that begins in early childhood, mainly manifested by different levels of impaired social interactions, speech stunting, communication deficits, restricted interest, and repetitive and rigid behavior.^1^^,^^2^ Epidemiological studies, conducted in a variety of groups around the world, have indicated ASD is increasingly common with a median prevalence of 1%.^3^^–^^6^ Since the first report on this disorder, ASD diagnostics has exclusively relied on behavioral observation.^7^ Even though such a diagnosis approach has been well accepted and achieved great success in clinical applications, there inevitably exist some shortcomings or limitations. For instance, it is difficult to make a diagnosis for children with high risk of ASD in their early childhoods, i.e., before age of 2 to 3, since during that period, their brains are fast developing and behaviors are changing; such unstable and inconsistent behaviors are not suitable for making the diagnosis. Besides, the complicated process of the diagnosis and the multiple behavioral assessments may result in children with ASD not being diagnosed in a timely fashion, which causes the children to miss the best time window for intervention or treatment. One attempt to solve this problem is to use brain imaging techniques, such as functional magnetic resonance imaging (fMRI), functional near-infrared spectroscopy (fNIRS), and electroencephalography (EEG), to uncover ASD-associated characteristics in brains of individuals with ASD. These noninvasive neuroimaging methods provide a safe way for studying the neural mechanisms of ASD and may open a new avenue for the diagnosis of ASD.

Among numerous studies on imaging ASD, a number of studies have revealed abnormalities in resting state functional connectivity (RSFC) in ASD patients,^8^^–^^12^ including children with ASD.^10^^,^^11^ RSFC refers to the temporal correlation of spontaneous brain activities among different regions of brain during the resting state,^13^^,^^14^ reflecting the functional synchronization, and coordination in these functionally related regions. Since abnormal RSFC has been demonstrated in autistic brains, RSFC might be served as a physiological characteristic for distinguishing the children with ASD from typically developing (TD) children. Several fMRI and fNIRS studies have consistently demonstrated under-connectivity between the bilateral temporal lobes (e.g., weak homotopic RSFC in temporal lobes) in ASD.^8^^,^^15^^–^^18^ The temporal lobes are involved in auditory processes such as spoken word recognition,^19^ the verbal and nonverbal aspects of social communication such as gaze and body movement,^20^^,^^21^ and the implicit emotion recognition related to empathizing ability.^22^ Therefore, the reduced RSFC between the bilateral temporal lobes may be associated with deficits in language and social interactions, which are core symptoms of ASD.

An important practical issue concerning the measurement of RSFC is the data acquisition time, i.e., how much time is required for recording the spontaneous brain activity in order to obtain reliable RSFC. In most RSFC studies using either functional fMRI or fNIRS, 5 to 10 min of acquisition time was usually adopted.^23^^–^^30^ This time range has been demonstrated to be suitable for acquiring fMRI-RSFC on young healthy adults since the measured RSFC has been observed to become stable after the acquisition time of about 5 min.^31^^,^^32^ On the other hand, resting-state fNIRS studies on young healthy adults showed that RSFC could become stable and reproducible after 1-min signal acquisition,^33^ whereas on healthy children, RSFC acquired in 1 min already showed high similarity to that measured with a longer acquisition time (e.g., 10 min), though it could take 7 min for RSFC to be accurate and stable.^34^

When using RSFC as a characteristic to differentiate between individuals with ASD and normal controls, short acquisition time for RSFC would make clinical implementation more feasible, particularly on populations that pose challenges to the time constraints of routine imaging, such as young children.^32^ Long data collection time require participants to keep still for a long time, which is difficult for the participants with the context of diseases, such as ASD. Therefore, if RSFC acquired from a relatively short measurement time can provide a reliable differentiation, it will reduce the burden for both experimenters and subjects. For healthy adults and children, the acquisition time required for accurately measuring RSFC has been examined and reported.^31^^–^^34^ However, for children with ASD it is not clear how long the acquisition time should be for accurately measuring RSFC, and what the minimal time is required for accurate differentiation between children with ASD and TD children. Since fNIRS is a noninvasive, portable, easy-to-use, and cost-effective optical brain imaging technique that has been widely utilized in studying RSFC,^35^ in the present work we used fNIRS to address this issue by investigating RSFC with different time windows ranging from short to long durations.

## 2 Method

### 2.1 Participants

Twenty-five children with ASD (9.3±1.4 years old) and twenty-two age-matched TD children (9.5±1.6 years old) participated in this study. In ASD group, there were 18 boys and 7 girls, all recruited from a local autism rehabilitation center. The TD group consisted of 18 boys and 4 girls. All children with ASD were diagnosed by experienced clinicians in hospital based on DSM-IV-TR,^36^ whereas TD children were healthy and had no history of any neurological or psychiatric disorders or physical injury. The intelligence quotient (IQ) values were assessed with Raven’s Standard Progressive Matrices Test.^37^ The mean score of IQ in ASD group was 91±15 and in TD group was 106±12. The difference in IQ was significant between the two groups (p<0.05). It has been demonstrated that intelligence performance may be related to functional connectivity between the frontal and other regions such as parietal, occipital, and limbic lobes,^38^^–^^41^ but there is no evidence showing that IQ is related to functional connectivity between the bilateral temporal lobes. Therefore, IQ could not be a factor affecting the RSFC in this study. Prior to the fNIRS data acquisition, the experimental procedure was clearly explained to the parents of children, and written consents were obtained from all of them. The study conformed to the recommendations of the University’s Ethical Review Board at South China Normal University.

### 2.2 Data Acquisition

**Fig. 1:**
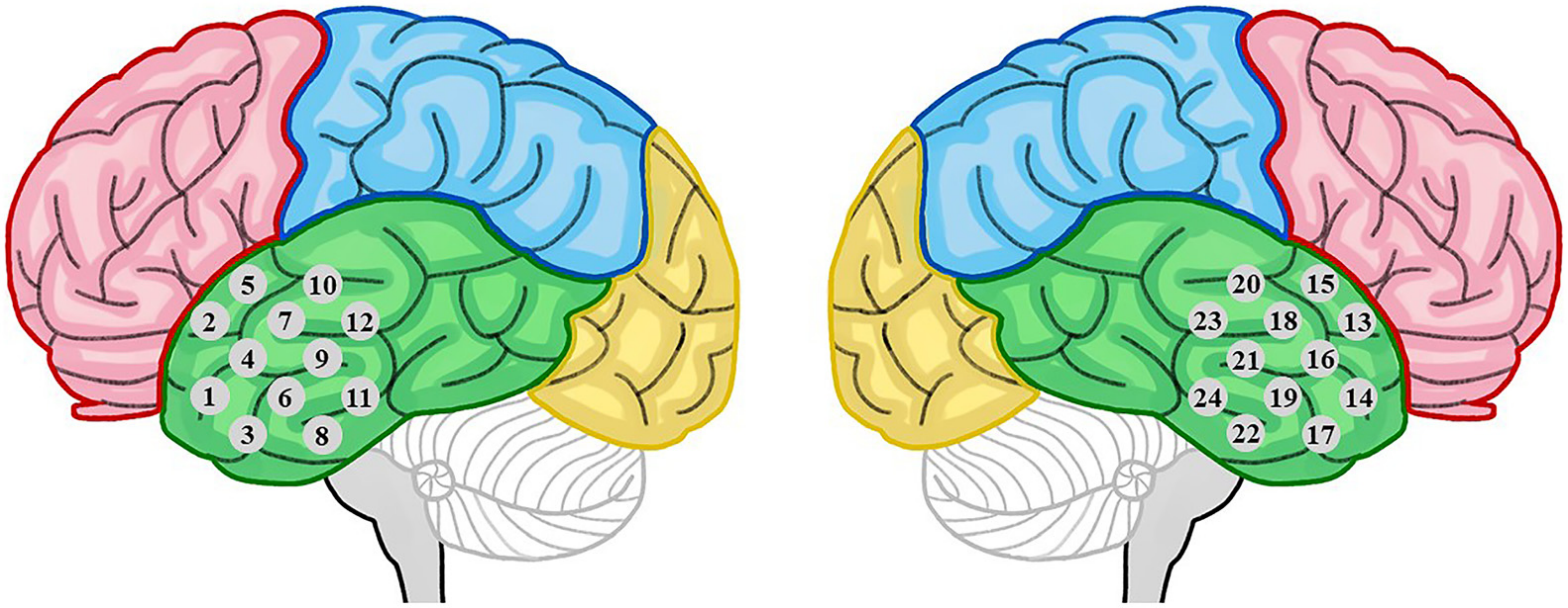
Schematic representation of fNIRS channel locations at the left and right temporal lobes. Each channel consisted of a source–detector pair.

During the measurement, subjects were sitting in a comfortable chair in a quiet and dark room. They were asked to keep their eyes closed, remain awake, and keep their bodies as motionless as possible. The hemodynamic fluctuations of each subject were recorded by a commercial continuous-wave fNIRS (FOIRE-3000, Shimadzu Corporation, Kyoto, Japan) working at 780, 805, and 830 nm wavelength. Ten light sources and 8 detectors were used, forming 24 fNIRS detection channels (a channel consisted of a source–detector pair). The sampling rate was 14.3 Hz (i.e., temporal resolution of 70 ms). The optodes were secured on the scalp by a headgear with a fixed source–detector distance of 30 mm, locating at the bilateral temporal lobes as shown in Fig. 1. The channel locations were determined according to the international 10-10 system. Spontaneous cerebral hemodynamic fluctuations of oxygenated hemoglobin (${\mathrm{HbO}}_{2}$), deoxygenated hemoglobin (Hb), and total hemoglobin HbT (${\mathrm{HbO}}_{2}+\mathrm{Hb}$), converted from the measured optical density via the modified Beer–Lambert law, were recorded for ∼8  min.

### 2.3 Data Analysis

In prior to the calculation of RSFC for each time window (e.g., 0.25, 0.5, 1.0, 1.5, 2.0 min,…), the time series of each hemodynamic variable (${\mathrm{HbO}}_{2}$ or Hb) was preprocessed for each channel, including a motion artifact correction by a wavelet algorithm in HOMER2 package,^42^^,^^43^ detrending by a second-order polynomial fit to remove slow drift, filtering with a band-pass (0.009 to 0.08 Hz) filter to eliminate most of the systemic hemodynamic components, such as those originated from cardiac cycles (∼1  Hz), respirations (∼0.2  Hz) and the Mayer waves (∼0.1  Hz), and obtain low-frequency signal, and an independent component analysis (ICA) approach for suppressing the global systemic interference in the signal.^17^

After data preprocessing, RSFC was calculated via the Pearson correlation coefficient for each mirrored channel pair locating symmetrically at the left and right hemispheres. Since there were 12 channels in each hemisphere as illustrated in Fig. 1, 12 correlation coefficients were obtained. To obtain the mean correlation coefficient between the left and right hemisphere, the 12 correlation coefficients could be averaged, which was viewed as the homotopic RSFC. However, there might be some poor channels with very low signal-to-noise level, which could lead to unreliable correlation coefficients. Such unreliable correlation coefficients could be identified by larger p values (for testing the hypothesis of no correlation), e.g., p>0.05. Therefore, in computing the homotopic RSFC, these unreliable correlation coefficients were excluded, and only those reliable correlation coefficients were taken into account for the average. To compute the homotopic RSFC, each reliable correlation coefficient r was converted to its z-value via Fisher’s r–z transform, and then we averaged the z-values to obtain the homotopic RSFC (indicated by z-value).

To investigate whether the difference in RSFC between the two groups (ASD or TD) was significant in each time window, two independent sample t-test was performed. For multiple comparisons, the false discovery rate (FDR) correction (i.e., FDR-corrected q value) was utilized. To restrain the type I (α) and type II (β) errors, the condition of q<0.05 and statistical power (1−β)>0.8 was considered as a criterion for significant level. For showing the discriminative ability of the RSFC, the receiver operating characteristic (ROC) curve and the AUC value were presented for each time window in which the RSFC had significant difference between the two groups.

## 3 Results

**Fig. 2:**
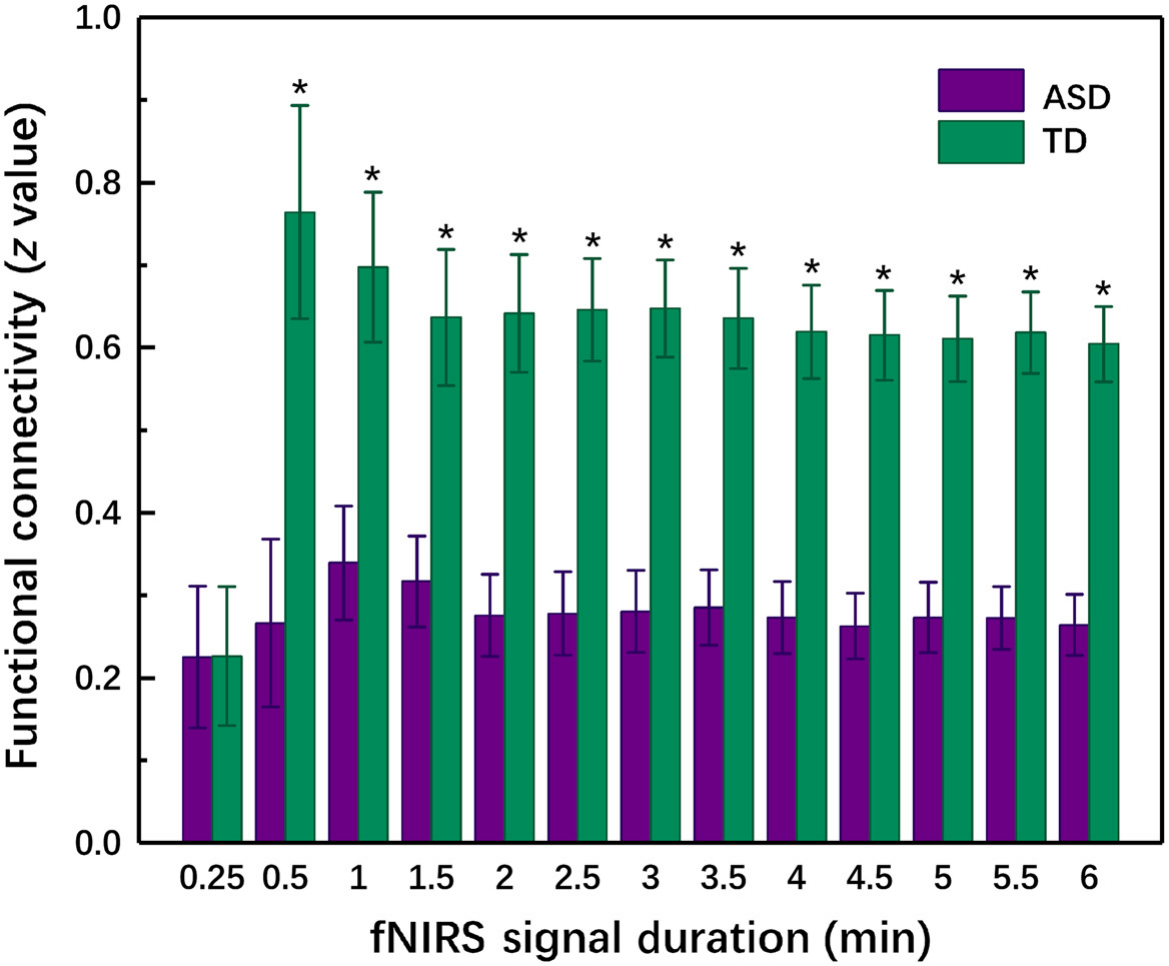
The homotopic ${\mathrm{HbO}}_{2}$-RSFC in the temporal lobes acquired with different acquisition times for TD and ASD groups. The asterisks indicate that the differences are statistically significant (i.e., FDR-corrected q<0.05 and the statistical power 1−β>0.8). The error bars are the standard error of mean.

To evaluate the difference in the homotopic ${\mathrm{HbO}}_{2}$-RSFC between the children with ASD and TD children, two samples t-test was performed for each time window. Table 1 shows the statistical parameters (i.e., FDR-corrected q value and statistical power 1−β) and AUC value for the ROC curve for different time windows. The difference in the ${\mathrm{HbO}}_{2}$-RSFC was significant between the children with ASD and TD children as long as the acquisition duration was longer than 0.5 min, which was also shown in Fig. 2.

**Table 1:** The statistical parameters of ${\mathrm{HbO}}_{2}$-RSFC for different time windows.

Time duration (min)	FDR-corrected q value	(1−β) value	AUC
0.25	0.9922	0.05	—
0.5	0.0033	0.86	0.72
1.0	0.0025	0.89	0.75
1.5	0.0020	0.91	0.76
2.0	<0.0001	0.99	0.82
2.5	<0.0001	1.00	0.83
3.0	<0.0001	1.00	0.84
3.5	<0.0001	1.00	0.85
4.0	<0.0001	1.00	0.85
4.5	<0.0001	1.00	0.88
5.0	<0.0001	1.00	0.88
5.5	<0.0001	1.00	0.89
6.0	<0.0001	1.00	0.89

**Fig. 3:**
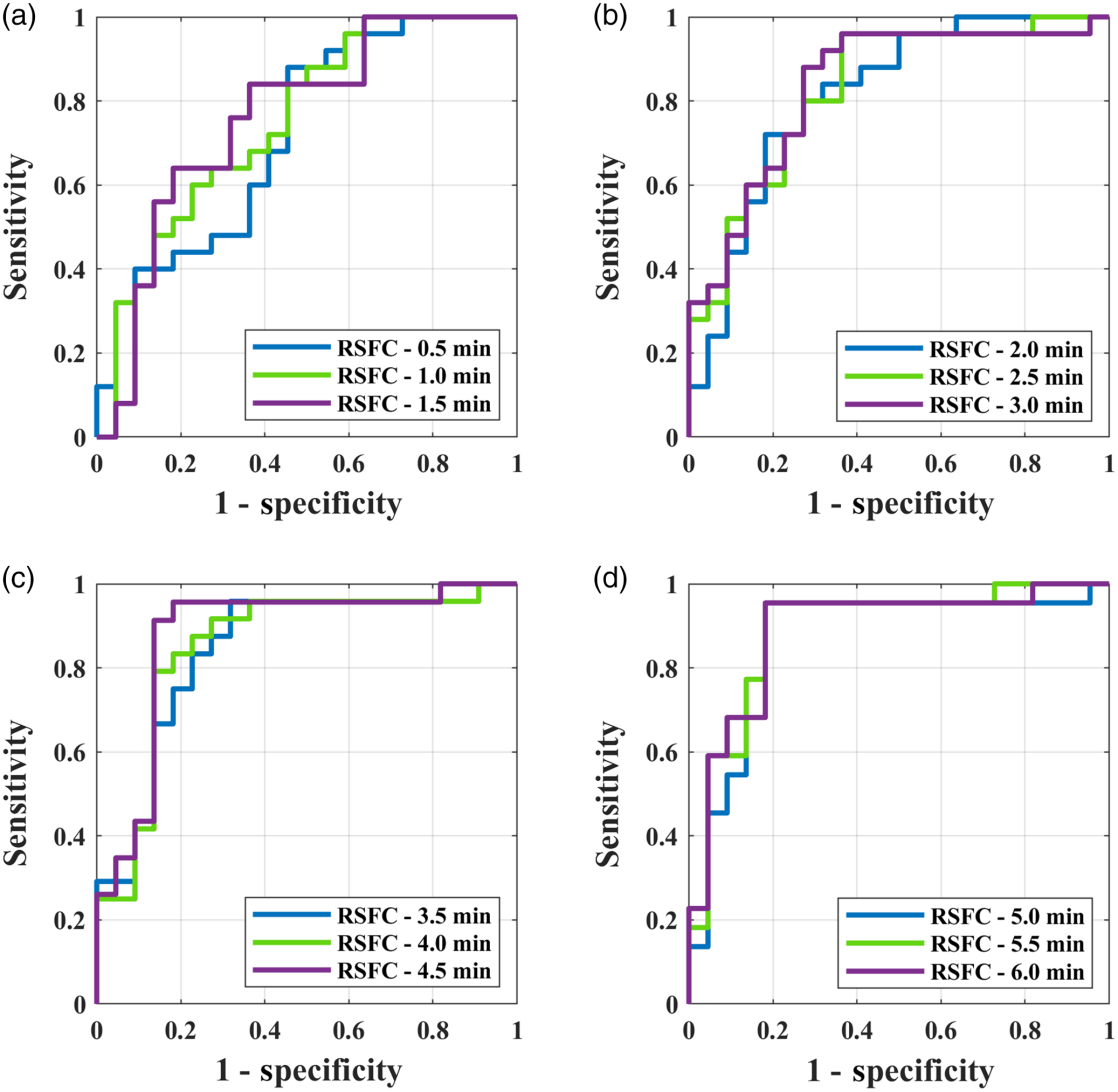
ROC curves were generated by using the ${\mathrm{HbO}}_{2}$-RSFC as a discriminative feature with different acquisition times in which the RSFCs were significantly larger in TD group than in ASD group. The AUC value was larger than 0.8 as long as the acquisition time was longer than 2 min. In each subfigure from (a)–(d), the ROC curves were plotted with 0.5 min increment in acquisition time.

To exhibit the discrimination ability of the RSFC obtained in different time durations, the ROC curves were drawn with ${\mathrm{HbO}}_{2}$-RSFC as a discriminative feature, as shown in Fig. 3. The ROC curve was generated by varying a criterion (i.e., the RSFC) to obtain the false-positive rate (1-specificity) and true-positive rate (sensitivity). An individual with RSFC larger than the criterion value was classified as TD, otherwise classified as ASD. The AUC value for the ROC curve indicates the differentiation ability between the two groups. The higher the AUC, the stronger the differentiation ability. From a very short, e.g., 0.5 min, to a long acquisition time e.g., 6 min, the AUC value gradually increased, e.g., from 0.72 to 0.89, indicating the differentiation ability increased with the time duration. Starting from 2 min to longer acquisition durations, the AUC values were consistently higher than 0.8, indicating good discrimination ability.^44^

For the homotopic Hb-RSFC, the two samples t-test showed that it would need a longer acquisition duration (i.e., >2.0  min) for the RSFC showing a significant (FDR-corrected q<0.05 and the power 1−β>0.8) difference between the two groups. In addition, the differentiation ability of Hb-RSFC was also worse than ${\mathrm{HbO}}_{2}$-RSFC, e.g., from 2- to 6-min acquisition time, the AUC value for Hb-RSFC ranged from 0.73 to 0.77, all <0.8. This was probably because of higher noise level in the Hb than in the ${\mathrm{HbO}}_{2}$ signal.

## 4 Discussion and Conclusion

Considering the negative influence of motion artifact on data quality, a wavelet algorithm was utilized in data analysis for both ASD and TD groups to correct the artifact induced mostly by the body or head movement. Children with ASD are usually believed to experience more movement than TD children during fNIRS data acquisition, since they may be more difficult to keep still. In this case, if the motion artifacts were not effectively removed from the fNIRS signals, the altered RSFC in ASD group might be due to the artifact, rather than the abnormal brain activity. To illustrate the alteration in RSFC in the present study was not from the motion artifact, we examined the coupling between ${\mathrm{HbO}}_{2}$ and HbT in each measurement channel via the correlation coefficients between them. Since in fNIRS measurement, head movement induces changes in ${\mathrm{HbO}}_{2}$ and HbT in the same direction, this may enhance the coupling between them. Therefore, if a motion artifact in the ASD group still made considerable or dominant contribution to the alteration in RSFC, the coupling between ${\mathrm{HbO}}_{2}$ and HbT could be stronger in the ASD group than in the TD group. However, we found the averaged coupling (averaged across all channels) in the ASD group was weaker (r=0.786) than the TD group (r=0.976), plausibly indicating the motion artifact was effectively suppressed by the wavelet algorithm in evaluating RSFC.

Several neuroimaging studies have investigated the acquisition duration for scanning resting-state brain for obtaining accurate and stable resting-state functional connectivity.^24^^,^^33^^,^^34^ fNIRS studies in both healthy children and adults^33^^,^^34^ have shown that RSFC measured with 1 min duration has already exhibited high similarity to those measured with longer acquisition times. This suggests shorter acquisition duration for measuring RSFC may be feasible in clinic applications, in particular scanning resting-state brains of young children who may not be able to keep their bodies motionless for a long time. The present fNIRS study on children with ASD and TD children revealed that the homotopic RSFC in temporal lobes measured with a very short acquisition time of 0.5 min had already showed significant difference between the two groups, which was previously observed with a longer acquisition time of 8 min. This indicated even in an unstable state due to short acquisition time, the RSFC still could be viewed as a discriminative index.

**Fig. 4:**
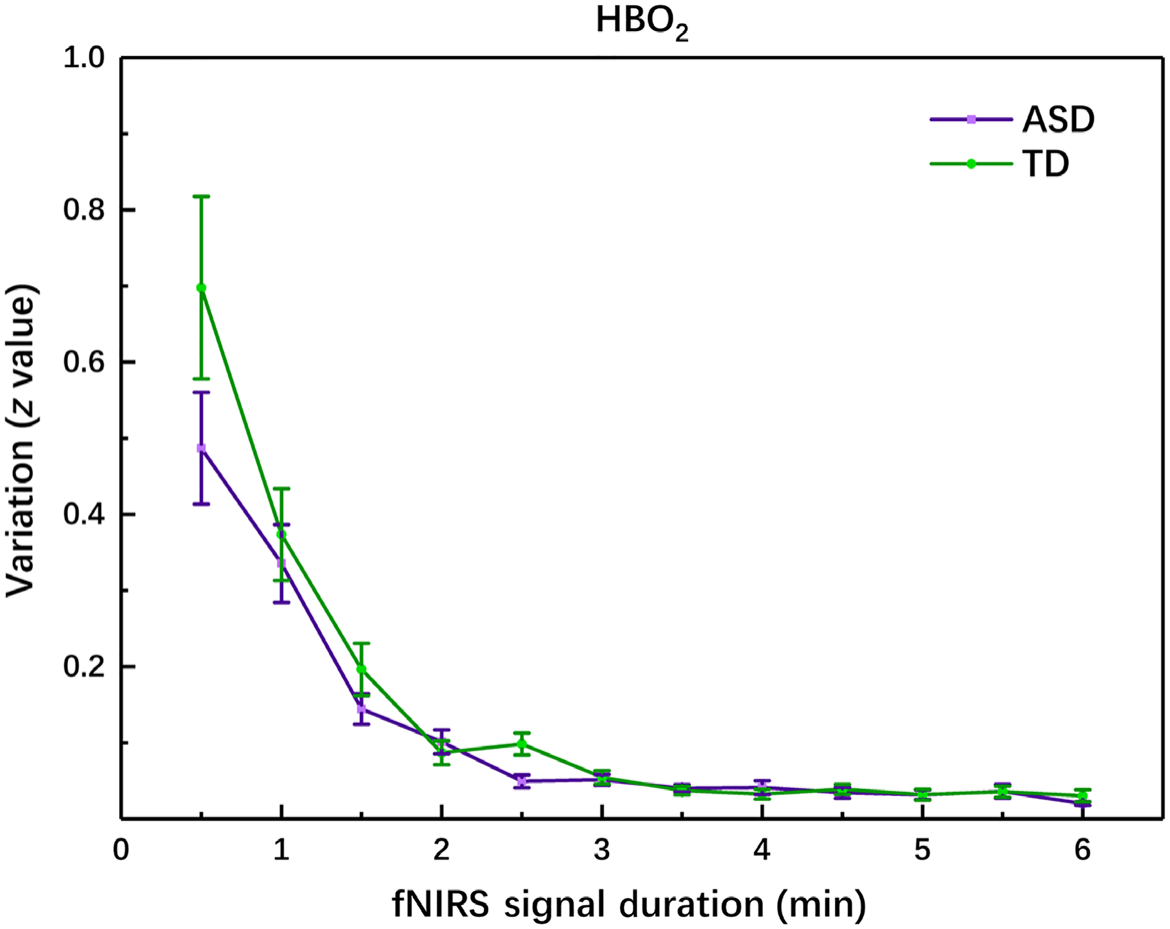
The variation between ${\mathrm{HbO}}_{2}$-RSFC acquired with two contiguous time durations (e.g., between 0.5 and 1 min, between 1 and 1.5 min, and so on) for ASD and TD groups. The variation showed almost no difference with acquisition time longer than 3 min in both ASD and TD groups. The error bars are standard errors of mean calculated in each group.

To investigate how the RSFC could vary with the acquisition duration in children with ASD and TD children, we calculated the variation in the homotopic RSFC between the two contiguous time durations. As shown in Fig. 4, the variation had almost no difference with acquisition time longer than 3 min in both groups, indicating the RSFC tended to be stable with acquisition time longer than 3 min. In addition, the two curves in Fig. 4 decayed nearly in the same way, indicating the RSFC for the two groups varied and converged with the acquisition time in almost the same way. This implied that if the RSFC was different for the two groups with a longer acquisition time, the difference was still there even with a short acquisition time. This might provide evidence that the two groups could be differentiated using the homotopic RSFC acquired even with a short time.

Investigation on how RSFC varies with acquisition time, and how long it may take for RSFC to become stable has been performed with fMRI in healthy adults,^31^^,^^32^ and fNIRS on healthy adults^33^ and healthy children.^34^ However, up to now, we have not found any published work reporting such a study on individuals with the context of diseases, particularly with ASD. Our study showed, as seen in Fig. 4, 3-min acquisition time might be long enough for accurately measuring RSFC in both children with ASD and TD children, since longer acquisition time (e.g., >3  min) could hardly lead to a more accurate measurement on RSFC. However, for the differentiation between ASD and TD, 2-min acquisition time for RSFC might be already long enough for achieving a good classification.

In contrast to the previous fNIRS studies^33^^,^^34^ in which the RSFC measured with a longer acquisition time (e.g., 10 min) was considered as a standard (or accurate) RSFC, and then the RSFC measured with a shorter time was compared to the standard one by calculating the correlation coefficient between them, in the present study we did not assume a standard RSFC; instead, we estimated the variation between the homotopic RSFC acquired with two contiguous time durations to find the trend of convergence. The variation was defined as the difference between the two values of homotopic RSFC. The variation in Fig. 4 showed a fast decay trend with the increase of acquisition time in both ASD and TD groups, and in particular, the two decay curves were almost same with acquisition time longer than 1.5 min. This could explain why even in a short acquisition time the difference between the two groups was still observable. In the previous neuroimaging studies, it has been observed individuals with ASD showed weaker RSFC between the bilateral temporal lobes acquired with a relatively longer acquisition time, e.g., 8 min.^15^^,^^17^^,^^18^ Since the varying trend of the RSFC with the increase of the acquisition time was nearly the same for ASD and TD groups, the difference in the RSFC between the two groups would be retained from short to long acquisition time. This could support our proposed hypothesis that the homotopic RSFC acquired with a short fNIRS signal acquisition time might also show a significant difference between children with ASD and TD children. Since the previous fNIRS study^33^ showed RSFC was reproducible after 1.0-min acquisition time in healthy adults, we evaluated the reproducibility of the RSFC measured in 1.0 min in the children (TD and ASD) via the analysis of variance (ANOVA) performed on RSFC values measured in many nonoverlapping 1-min windows, and found there was no significant difference (p=0.93) between each other, implying that the spontaneous fNIRS signal acquisition with a short time duration such as 1.0-min could lead to a stable homotopic RSFC in children.

The present study focused on investigating the fNIRS homotopic RSFC acquired with a short acquisition time to characterize children with ASD. We found even with a very short acquisition duration of 0.5 min, the RSFC already exhibited a significant difference between the children with ASD and TD children (Table 1). With the increase of the acquisition duration, the differentiation ability of the RSFC increased, which was manifested by the increase in AUC value of the ROC curve. When the acquisition time was longer than 2.0 min, the AUC value was consistently larger than 0.8, indicating a good differentiation could be achieved with the RSFC as a discriminative feature. In several resting-state fMRI studies using functional connectivity (FC),^45^^,^^46^ entropy,^47^ and phase synchrony (PS)^48^ as discriminative features to differentiate between individuals with ASD and TD controls, the AUC values achieved were 0.81 to 0.99. For example, AUC (PS) = 0.81,^48^ AUC (sample entropy) = 0.89,^47^ AUC (Slow-4 and -5 FC) = 0.86,^45^ and AUC (dorsal attention networks) = 0.99;^46^ In an fNIRS study using narrowband (0.01 to 0.02 Hz) RSFC, the AUC value achieved was 0.87.^49^ In these studies, the acquisition durations were generally longer, e.g., 6.0 to 10.0 min. As seen in Table 1, a longer acquisition time could lead to a higher AUC value, implying a better differentiation. However, our experience shows when measuring resting-state brain activity, it is usually difficult for children with ASD, in particular low-functioning ASD, to keep quiet and motionless even for a few minutes, e.g., 3 to 5 min. Therefore, reducing the acquisition time, while keeping the recorded data good enough for making a good differentiation is critical for imaging ASD. Our results showed that 2-min time duration for measuring resting-state brain might be marginally acceptable for achieving a good differentiation based on the homotopic RSFC.

A limitation of this study was the fNIRS signals measured were inevitably mixed with interferences from scalp and skull. Since the fNIRS system used was not equipped with short source-detector channels, we could not use a short channel regression approach to effectively eliminate these interferences, especially those being locally different, which could not be suppressed by the ICA approach. Therefore, in this study we assumed there was no difference between the two groups (ASD and TD) in RSFC originated from extracerebral tissues.

In conclusion, children with ASD showed significant reduction in fNIRS homotopic RSFC between the bilateral temporal lobes, which could be observed even in a short acquisition time of 0.5 min. Using the RSFC as a discriminative feature to differentiate between children with ASD and TD children, 2.0 min might be a minimal acquisition duration for achieving a good differentiation, though the accuracy of the differentiation could be improved by a longer acquisition duration.

Thanks to the advantages such as a noninvasiveness, portability, relatively low sensitivity to head movement, no need for a special measuring environment, and being easy to use, fNIRS may be a suitable imaging modality for investigating characteristics in children with ASD. With more characteristics associated with ASD revealed, the sensitivity and specificity for the differentiation between children with ASD and TD children could be improved, which might render fNIRS a useful tool for ASD screening in children with a high risk of ASD, even in an outpatient or community clinic. For the screening, short acquisition time may be more feasible in clinics.
